# BIRC3 and BIRC5: multi‐faceted inhibitors in cancer

**DOI:** 10.1186/s13578-020-00521-0

**Published:** 2021-01-07

**Authors:** Raffaele Frazzi

**Affiliations:** Laboratory of Translational Research, Azienda Unità Sanitaria Locale - IRCCS di Reggio Emilia, Viale Risorgimento 80, Reggio Emilia, Italy

**Keywords:** IAP proteins, *BIRC3*, *BIRC5*, Chronic lymphocytic leukemia, Smac‐mimetics

## Abstract

**Background:**

The evasion from apoptosis is a common strategy adopted by most tumors, and inhibitors of apoptosis proteins (IAPs) are among the most studied molecular and therapeutic targets. *BIRC3* (cellular IAP2) and *BIRC5* (survivin) are two of the eight members of the human IAPs family. This family is characterized by the presence of the baculoviral IAP repeat (BIR) domains, involved in protein-protein interactions. In addition to the BIR domains, IAPs also contain other important domains like the C-terminal ubiquitin-conjugating (UBC) domain, the caspase recruitment (CARD) domain and the C-terminal Ring zinc-finger (RING) domain.

**Main body:**

*BIRC3* and *BIRC5* have been characterized in some solid and hematological tumors and are therapeutic targets for the family of drugs called “Smac mimetics”. Many evidences point to the pro-survival and antiapoptotic role of *BIRC3* in cancer cells, however, not all the data are consistent and the resulting picture is heterogeneous. For instance, *BIRC3* genetic inactivation due to deletions or point mutations is consistently associated to shorter progression free survival and poor prognosis in chronic lymphocytic leukemia patients. *BIRC3* inactivation has also been associated to chemoimmunotherapy resistance. On the contrary, the progression from low grade gliomas to high grade gliomas is accompanied by *BIRC3* expression increase, which bears relevant prognostic consequences. Due to the relationship between *BIRC3*, MAP3K14 and the non-canonical NF-kB pathway, *BIRC3* inactivation bears consequences also on the tumor cells relying on NF-kB pathway to survive. *BIRC5*, on the contrary, is commonly considered an anti-apoptotic molecule, promoting cell division and tumor progression and it is widely regarded as potential therapeutic target.

**Conclusions:**

The present manuscript collects and reviews the most recent literature concerning the role played by *BIRC3* and *BIRC5* in cancer cells, providing useful information for the choice of the best therapeutic targets.

## **Background**

**Fig. 1:**
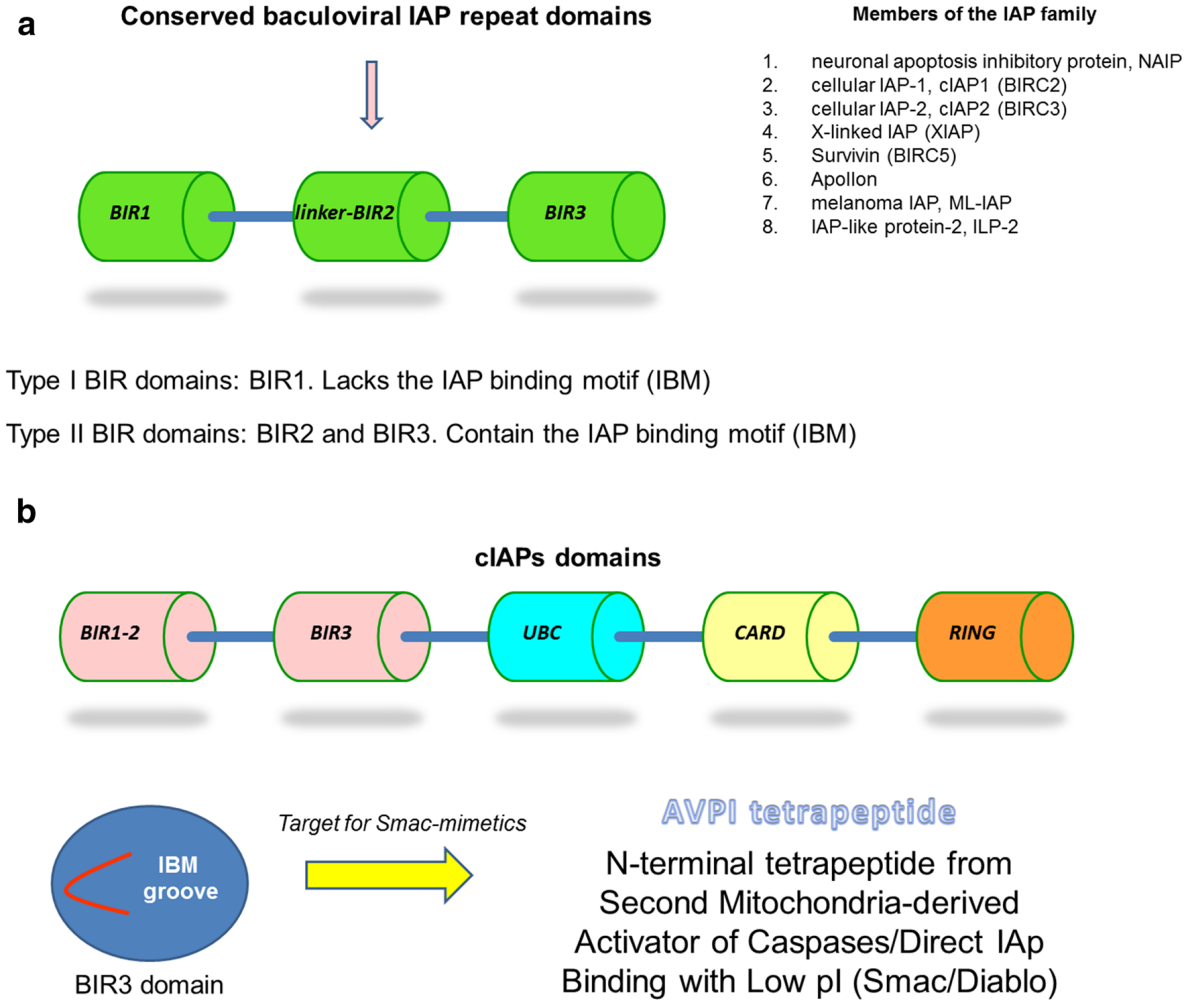
The structure and composition of inhibitory of apoptosis proteins (IAPs) family members. **a** The three BIR domains characterize the members of the IAPs family, summarized in the list at the right corner. BIR1 does not contain the IAP binding motif (IBM), while BIRC2 and BIRC3 do contain the IBM and, thus, can interact with effector caspases and with Smac/Diablo. **b** Cellular IAPs (cIAPs) contain a caspase recruitment (CARD) domain in addition to the three BIR domains, the ubiquitin-conjugating (UBC) domain and the C-terminal Ring zinc-finger (RING) domain. The IBM groove is the one responsible for the binding of the N-terminal tetrapeptides

Apoptosis is a cell death pathway that is physiologically adopted by human cells in response to death signals. It may follow either an intrinsic (mitochondrial) or an extrinsic (death receptor-mediated) pathway [1]. The evasion from apoptosis is a common strategy adopted by most tumors, and inhibitors of apoptosis proteins (IAPs) are among the most studied molecular and therapeutic targets. *BIRC3* (cellular IAP2) and *BIRC5* (survivin) are two of the eight members of the human IAPs family [2, 3]. This family is characterized by the presence of the baculoviral IAP repeat (BIR) domains, involved in protein-protein interactions. In addition to the BIR domains, IAPs also contain other important domains like the C-terminal ubiquitin-conjugating (UBC) domain, the caspase recruitment (CARD) domain and the C-terminal Ring zinc-finger (RING) domain [2, 4] (Fig. 1).

**Fig. 2:**
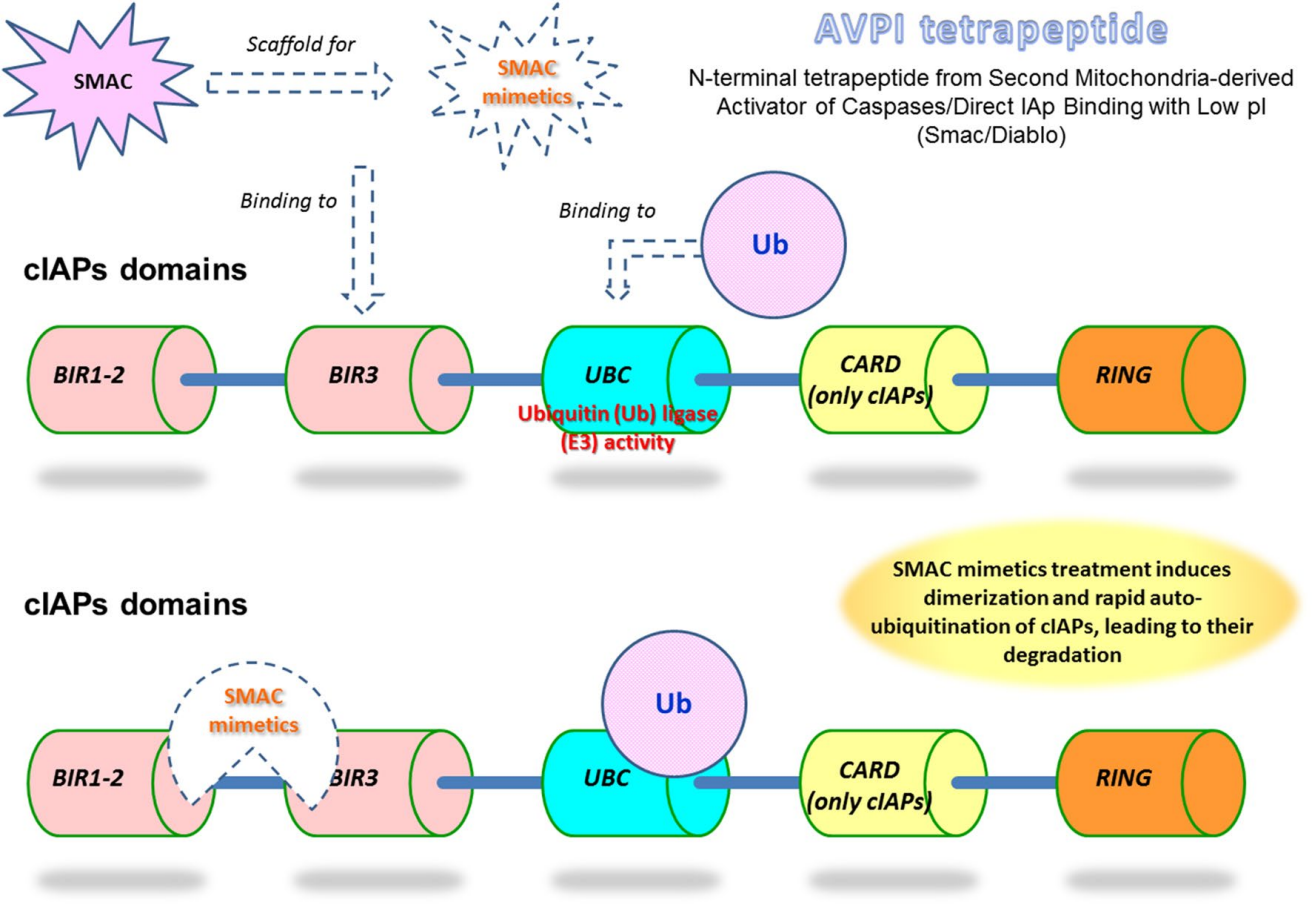
SMAC mimetics activity on cIAPs. SMAC mimetics bind to BIR3 domains and induce homo-dimerization. Ubiquitin is recruited and leads to cIAP degradation

The strategies aimed at restoring the apoptotic processes in cancer cells led to the discovery of IAPs antagonists. X-linked IAP (XIAP/*BIRC4*) is one of the best characterized and is a target for therapeutic intervention. The natural inhibitor of XIAP is the Second Mitochondria-derived Activator of Caspases/Direct IAp Binding with Low pI (SMAC/Diablo), a N-terminal tetrapeptide who inspired IAPs antagonists called “SMAC mimetics” [5]. Great interest raised around SMAC mimetics as novel active compounds in the fight against cancer. These molecules are designed to block the IAPs activity, including *BIRC3* and *BIRC5*, thus promoting apoptosis (Fig. 2). SMAC mimetics have been tested as single agents and in combination to proteasome inhibitors, leading to promising results. SMAC mimetics are also under investigation for the therapy of diffuse large B-cell lymphoma (DLBCL) and chronic lymphocytic leukemia (CLL) [4, 6].

Genetic aberrations also represent a mechanism for IAPs disregulation. It has been recently reported that 11q22.1-q22.2 locus amplification represents a marker for poor clinical outcome and metastasis progression in oral squamous cell carcinoma (OSCC) [7]. This locus codes for cellular IAP1 (cIAP1) and cellular IAP2 (cIAP2) (*BIRC2* and *BIRC3*, respectively) and its amplification is also associated to lymph node metastasis and radioresistance insurgence in OSCC, strongly supporting the oncogenic function of these two IAPs in this type of malignancy [8].

The laboratory and pre-clinical evidences collected thus far are not entirely concordant though. IAPs are commonly considered pro-oncogenic proteins, associated to cancer cell evasion from death mechanisms, progression of cell cycle and proliferation. However, several clinical studies published in recent years reported an unfavorable contribution of *BIRC3* genetic inactivation or downregulation in cancer patients. These evidences suggest that a more complex scenario is actually regulating the expression and modulation of these genes and needs to be taken into account when designing therapeutic approaches.

The present manuscript aims at collecting and describing the most recent evidences concerning the role of the two IAPs *BIRC3* and *BIRC5* focusing on cancer, in order to underline the common characteristics and to shed light on the main controversies.

## Mechanisms of evasion from apoptosis

Cancer cells display a number of different mechanisms in order to evade apoptosis. In particular, the activation of anti-apoptotic systems allows cancer cells to escape this program leading to uncontrolled proliferation resulting in tumor survival, resistance to therapies and recurrence of cancer. Some of the most relevant may be subdivided in these major groups:

2.1. *Dysregulation of anti-apoptotic BCL-2 members*.
2.2. *Inhibitors of caspases*.
2.3. *Involvement of autophagy*.
2.4. *Involvement of Heat shock protein 90 (Hsp90)*.
2.5. *Involvement of nuclear transport regulation*.

### Dysregulation of anti-apoptotic BCL-2 members

The members of the B-cell lymphoma 2 (*BCL-2*) family regulate the mitochondrial pathway of apoptosis. This family includes both anti-apoptotic and pro-apoptotic proteins and is characterized by the presence of one or more BCL-2 Homology (BH) domains (BH1–BH4) [1]. The overexpression of the antiapoptotic *BCL-2* family members compared to the normal counterpart is a common mechanism for apoptosis resistance of cancer cells in several tumors, including mature B-cell neoplasms [9, 10]. Several drugs that entered into the clinic in recent years are targeted towards BCL-2 in overexpressing tumors. These are BH3 mimetics (they mimic the physiological activity of BCL-2 antagonists) and kill cancer cells by targeting their survival mechanisms. Venetoclax (ABT-199) is, for instance, the first in class orally bioavailable BCL-2 selective BH3 mimetic that binds BCL-2 while sparing BCL-XL and MCL-1 [11].

### Inhibitors of caspases

Inhibitors of apoptosis (IAP) proteins are a class of apoptosis regulators that perform several functions, including the control of survival and cell death by regulating crucial factors in signaling events such as caspase activation and NF-κB signaling [12]. IAPs were initially discovered in baculoviral infected SF-21 insect cells and later on in many organisms, including humans [13]. The IAP-binding motif (IBM) is the portion required for the interaction with the N-terminus of some caspases and IAP antagonists. Changes in the aminoacidic composition of IBM alter the binding selectivity of different IAPs [14].

### Autophagy

The autophagic process is a physiological mechanism aimed at maintaining cell homeostasis through lysosomal degradation of unnecessary or damaged cellular components. It is commonly considered a cell survival mechanism. Thus, in an attempt to maintain the intracellular balance after chemotherapy, autophagy may represent a further mechanism for cancer cells to escape cell death [10, 15, 16]. Autophagy has been observed to protect cancer cells from apoptosis upon treatment with some anticancer drugs and it may drive the acquisition of chemoresistance [17–19]. Among the most recent evidences supporting this phenomenon, there is the relevant finding that major histocompatibility complex class I (MHC-I) surface molecules expressed by pancreatic cells can be degraded through autophagy [20]. Pancreatic ductal adenocarcinoma (PDAC) is a very aggressive form of cancer, resistant to most therapies including immune checkpoint blockade. Yamamoto et al. [20] demonstrated that PDAC display a reduced MHC-I surface expression, while accumulating this molecule in the autophagosomes and lysosomes. In an experimental model of syngeneic host mice, the autophagy inhibition restores the MHC-I surface levels leading to improved antigen presentation, enhanced anti-tumor T cell responses and reduced tumor growth. Furthermore, autophagy may trigger a form of programmed cell death (type II programmed cell death) induced by an excess of cellular stress [21]. Thus, autophagy is also connected to cell death mechanisms, including apoptosis, and should be considered also a cellular death promoter under specific circumstances.

### Heat shock protein 90 (Hsp90)

Hsp90 is a highly expressed chaperone molecule that plays anti-apoptotic functions by chaperoning non-mutated and mutated kinases and cytosolic anti-apoptotic factors. This chaperoning activity leads to proliferation, migration and metastasis enhancements, that is to say Hsp90 may drive tumor progression [10]. The resistance to Tyrosine kinase inhibitors in non-small cell lung cancer (NSCLC) is mediated, for instance, by Hsp90. Hsp90 inhibitors have been demonstrated to preferentially deplete mutated EGFR, with a consequent suppression of p-Akt and induction of cell death [22]. NSCLC targeted therapy had also been demonstrate to benefit of Hsp90 inhibition in tumors harboring EGFR mutations [23, 24]. Notably, *BIRC5* (survivin) is a client protein of Hsp90 chaperone and results downregulated upon Hsp90 pharmacological inhibition in cancer cells [25, 26].

### Nuclear transport regulation

The nuclear pore complexes (NPC) became attractive therapeutic targets because the aberrant expression of the constituent proteins has been consistently observed in different cancers and has been linked to apoptosis resistance [27]. The subcellular localization of apoptosis inducers is crucial and tumor suppressors usually reside in nucleus where they exert their function by binding to DNA in a sequence-specific fashion leading to modulation of gene expression and assessing the integrity of the genome [28]. The inhibition of nucleo-cytoplasmic transport is key in order to keep the cell cycle regulators and oncosuppressors within the nucleus and inhibit cancer cell growth. Studies of synergism between venetoclax (ABT-199, a selective BCL-2 inhibitor) with selective inhibitors of nuclear export (SINE) yielded in vitro and in vivo promising results in acute myeloid leukemia (AML) and diffuse large-B cell lymphoma (DLBCL) models [29].

## The role of BIRC3 during evasion of cancer cells from apoptosis

Here are presented some updated evidences concerning the tumors where a role for cIAP2 (*BIRC3*) emerged as pivotal for prognosis and during the insurgence of therapy resistance.

Chronic lymphocytic leukemia, gliomas/glioblastomas and breast cancer are considered. The analysis of the experimental evidences point to an apparent paradox of this cIAP, since in many instances *BIRC3* plays a role of tumor suppressor, its deficiency being associated to poor prognosis and insurgence of therapy resistance (please, see Table 1).

**Table 1:** *BIRC3* and *BIRC5* roles in different cancers

Tumor type	Function	References
*BIRC3*		
Oral squamous cell carcinoma (OSCC)	Pro-oncogenic: poor prognosis, metastasis, radioresistance	[7, 8]
Chronic lymphocytic leukemia (CLL)	Oncosuppressive: disruptions predict poor prognosis, inferior outcome, chemoresistance. Neg. regulator of the non-canonical NF-kB pathw.	[36–45, 49]
Chronic lymphocytic leukemia (CLL)	Pro-oncogenic: higher expression in leukemia cells, downregulated by SMAC-mimetics	[50]
Mantle-cell lymphoma (MCL)	Oncosuppressive: mutations activate the non-canonical NF-kB pathw.	[51–54]
Glioma, glioblastoma (GBM)	Pro-oncogenic: gene expression inversely correlates to survival and therapy resistance. Higher expression in HGG	[3, 12, 56]
Breast cancer	Pro-oncogenic: antiapoptotic, chemoresistance	[57–62]
Breast cancer	Oncosuppressive: high expression correlates to drug sensitivity	[2]
*BIRC5*		
Lung, pancreatic, breast, ovarian, brain, colon cancer	Pro-oncogenic	[63–65]
B-cell acute lymphoblastic leukemia, B-cell lymphoma and T-cell leukemia/lymphoma	Pro-oncogenic	[66–68]
Hepatocellular carcinoma (HCC)	Pro-oncogenic: high expression correlates to lower survival	[70]
Gastrointestinal stromal tumors (GIST)	Pro-oncogenic: high expression correlates to lower survival	[71]
Prostate cancer	Pro-oncogenic: high expression correlates to p53 mutations and metastases. Cytoplasmic localization associates to an aggressive disease	[72, 74]
Gioma, astrocytoma, glioblastoma, medulloblastoma	Pro-oncogenic: anti-apoptotic function. High expression correlates to lower short-term and long-term survival. Overexpression increases chromosomal aberrations	[64, 75–79]
Colorectal cancer, ALL, melanoma, glioblastoma	Pro-oncogenic: silencing and inhibition leads to chemo- and radiosensitization	[80–84]

### Chronic lymphocytic leukemia and lymphomas

CLL/small lymphocytic lymphoma is a common B-cell malignancy characterized by a highly variable clinical course. CLL is the most common leukemia of adults in Western countries and the third most common malignancy of B-cell origin in the United States [30–32].

It is now accepted that CLL is characterized by a significant amount of chromosomal abnormalities, which were discovered and described during the 2000’s by fluorescence in situ hybridization (FISH) and sequencing, and led to a FISH-based hierarchical prognostic model that is valid, to some extent, even today [33–36].

In addition to deletion 17p (del17p), del11q, trisomy 12 and del13q, several point mutations have been described and may coexist with the deletion on the other allele (e.g. *TP53*, *ATM*, et al.). Among the most relevant point mutations for their predictive or prognostic value are the ones within the *BIRC3* gene [36].

Even though *BIRC3* mutation or deletion has been reported in about 3–7% of the CLL cases, its disruption predicts poor prognosis and represents an independent risk factor [37, 38]. *BIRC3* deletions and mutations are recognized as rare albeit unfavorable events for CLL patients. A significant contribution to the research in mutation landscape of CLL reported a few years ago that *BIRC3* abnormalities associate to an inferior outcome in the LLC0405 protocol [39]. The results on the independent negative predictive value of *BIRC3* were also confirmed in a large, comprehensive study where the mutational and cytogenetic analysis were integrated. The highest-risk group emerging from this analysis was the one harboring *TP53* and/or *BIRC3* abnormalities and displayed a significantly lower 10-years survival rate compared to the low-risk group [40].

It is widely accepted that deletion of 11q (del11q-) is a relevant aberration in CLL and is associated to unfavourable prognosis [41]. Del11q is a recurrent karyotypic abnormality acquired by patients with progressive CLL disease. Initial karyotypic and FISH studies were complemented by genotypization of CLL patients, leading to the discovery that del11q is monoallelic, often large and includes a minimal deleted region encompassing *ATM* gene [37]. The minimal deleted region often includes also *BIRC3*, located on the 11q22.2 band, *in cis* with *ATM*. According to the most recent literature on CLL, *BIRC3* inactivation identifies a subgroup of patients with very aggressive disease [42]. Furthermore, the patients with a biallelic lesion of *BIRC3* (del and mut) were associated to a significant shorter time to first treatment when compared to *BIRC3*-del/wt or wild type patients [42].

*ATM* is involved in DNA damage repair whereas *BIRC3* is a negative regulator of non-canonical NF-kB signaling. *BIRC3* deletion occurs in 83% of del11q cases and always coexists with *ATM* deletion, as demonstrated by the CLL4 study [37].

Concerning the response to therapies, it is known that *BIRC3* inactivation is associated to fludarabine-chemoresistance and to adverse prognosis in a large cohort of chemotherapy-treated CLL patients [43]. Furthermore, a target re-sequencing of 22 genes of the patients enrolled in the UK LRF CLL4 study confirms that bi-allelic *BIRC3* lesions (del and mut in the same patient) are an independent marker of inferior progression free survival (PFS) and overall survival (OS) [44]. This large, multi-centrical study (n = 499 patients) reports a detailed distribution of the mutational landscape of CLL disease after target re-sequencing. Del11q cases are the second most frequent copy number alteration (CNA) and the third most frequent genetic alteration [44]. *BIRC3* point mutations can also co-occur with del11q and *NOTCH1* point mutations. Both co-occurrences are statistically significant by Fisher’s exact test. At variance with this association, *BIRC3* point mutations do not occur with *ATM* point mutations, but associate to del11q. An integrated analysis of *ATM* and *BIRC3* mutations in a context of del11q patients revealed that biallelic *BIRC3* mutations associate to shorter PFS and OS by Kaplan Meier plots, confirming an unfavorable prognostic value reported also by Raponi et al. [42, 44].

*BIRC3* contains three adjacent BIR domains, each made of about 70 amino acids, whose fold is stabilized by a zinc atom that is coordinated by one histidine and three cysteine residues [4, 43]. In order to better understand the correlation between *BIRC3* gene inactivation and worse prognosis, we first of all took a look at what types of mutations occur within *BIRC3* gene. There exist two clusters of mutations in the coding region, as just reported in the analysis by Diop et al. [43]. These two mutation hotspots are located between amino acids 367–438 and amino acids 537–564 (UBC domain and RING domain respectively). They are frameshift and stop codons mutations, eventually leading to the inactivation/truncation of the C-terminal RING domain of the protein (Fig. 1). The RING domain is necessary for the interaction with the E3 ubiquitin ligase that leads to ubiquitin-mediated proteasomal degradation of MAP3K14 [45]. MAP3K14 is the central kinase of the NF-kB non-canonical pathway. It is therefore correct to consider the actual *BIRC3* mutations as NF-kB-activating, by MAP3K14 stabilization.

The recent work by Diop et al. [43] further demonstrates that *BIRC3* mutations confer, at least partially, resistance to fludarabine treatment on primary CLL samples. In the same experimental set also *TP53* mutant and wt CLL samples were treated, confirming the resistance and sensitivity to treatment respectively. In this same study, the prognostic significance of a panel of the most frequent mutations was assessed in a total of 287 CLL patients who received first-line fludarabine-cyclophosphamide-rituximab (FCR). The univariate analysis adjusted for multiple comparisons unveils that just *TP53* and *BIRC3* mutations associate to a significantly shorter PFS. These two mutations were consistently associated to a lower complete response rate at the end of the FCR therapy.

**Fig. 3:**
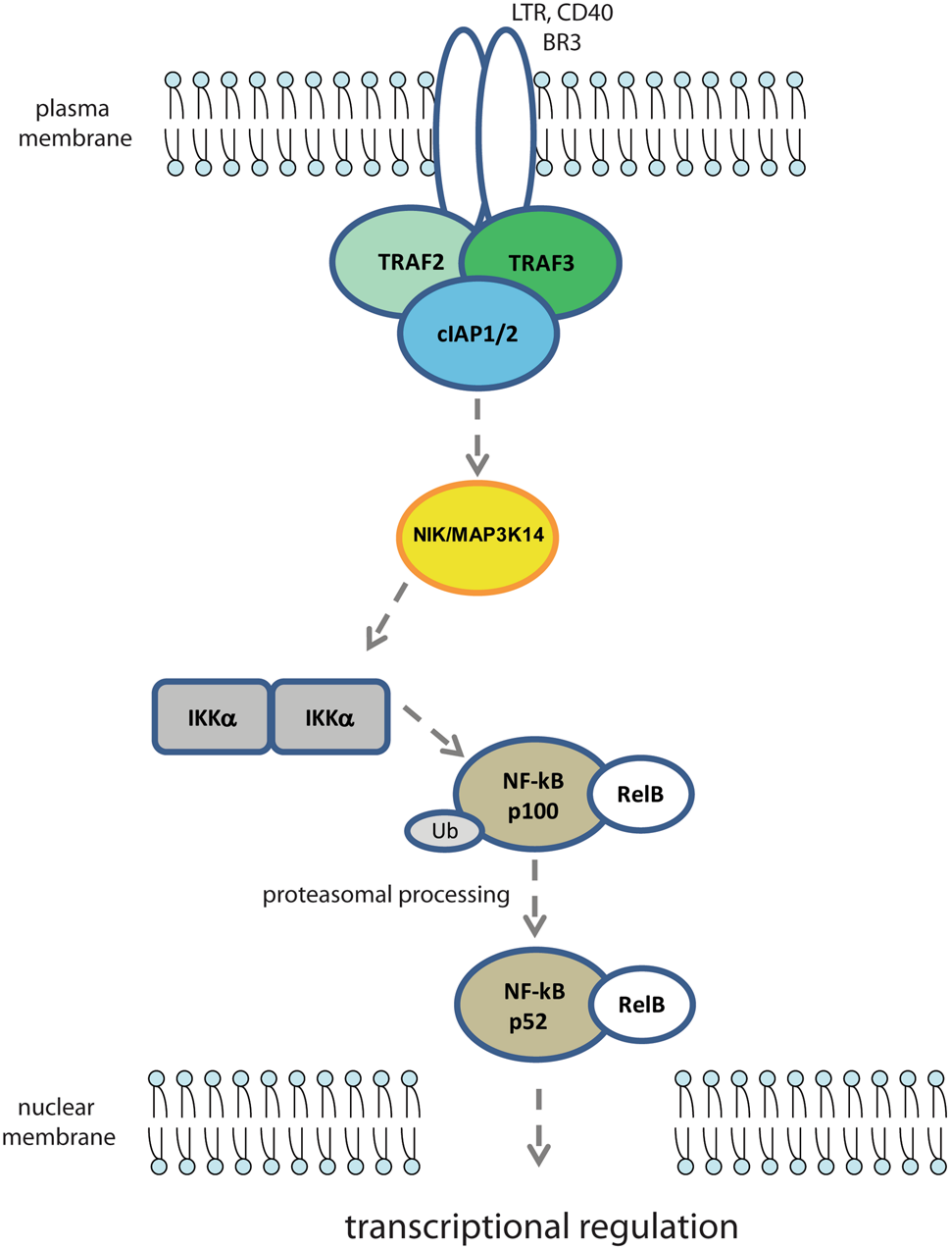
Schematic representation of the pathway involving the non-canonical NF-kB activation through NF-kB-inducing kinase (NIK/MAP3K14), modulated by cIAPs

Collectively, the data summarized above point to a tumor suppressive function of *BIRC3*, likely through the cooperation with other mediators. This function is consistent with the inhibitory activity exerted on the non-canonical NF-kB pathway, which is active in the CLL cells and enhanced by *BIRC3* deficiency (Fig. 3).

These evidences also do not fit with the concept that *BIRC3* is a promoter of malignancy due to its apoptosis-inhibitor capabilities (it is a member of the IAP family). If this were the case, a *BIRC3* deficiency would facilitate the apoptotic process of CLL cells, especially upon chemo-immunotherapy. *BIRC3* is also a target for SMAC mimetics directed not only towards XIAP but also towards cIAP1 (*BIRC2*) and cIAP2 (*BIRC3*) [46, 47]. Of note, it has been reported a functional redundancy between different IAP members, namely cIAP1, cIAP2 and XIAP [48]. These overlapping functions could represent an escape mechanism of cancer cells after the administration of SMAC mimetics and a strategy leading to drug resistance.

Further evidences pointing to the tumor-suppressor role of *BIRC3* in CLL arise from the characterization of IBTKα in CLL primary samples [49]. IBTKα is an inhibitor of the Bruton tyrosine-kinase (BTK) pathway associated to CLL stage and progression. Some evidences reports IBTKα expression increase with the Binet stage of the disease. *BIRC3* is a downstream target regulated by this inhibitor and its expression anti-correlates with IBTKα-silencing, supporting its role as a tumor suppressor [49].

However, it has to be mentioned that not all the experimental data available on CLL are consistent. A recent pre-clinical research demonstrates how XIAP, cIAP1 and cIAP2 are more expressed by CLL cells compared to normal lymphocytes [50]. A SMAC mimetic targeting the IAP’s BIR3-domain was able to induce apoptosis of CLL cells through a specific XIAP- and IAP-degradation. This work also showed how XIAP and cIAP2 high expression in microenvironment-like experiments was downregulated by SMAC mimetics, suggesting how *BIRC3* can be considered an oncogene and a therapeutic target [50].

A focus on mature B-cell neoplasms reveals how *BIRC3* mutations are common to another lymphoid malignancy: mantle-cell lymphoma (MCL) [51]. Ibrutinib inhibits BTK pathway and, as such, it represents a promising therapeutic options also for MCL patients [52]. Primary ibrutinib resistance may arise due to mutations in the B-cell receptor (BCR) pathway causing its constitutive activation (e.g. BTKC481S mutation). These eventually lead to constitutive activation of the canonical NF-kB pathway. It has been demonstrated though that MCL-cell lines rely either on the canonical (BCR-BTK‐NF-kB) or non-canonical (MAP3K14‐NF-kB) NF-kB pathways and may develop ibrutinib resistance. Mutations in the *TRAF2*, *TRAF3, BIRC3* and *MAP3K14* genes may lead to a constitutive activation of the non-canonical NF-kB pathway through MAP3K14 stabilization (Fig. 3). Furthermore, *TRAF2*, *BIRC3* and *MAP3K14* were recurrently mutated in approximately 17% of a cohort of 165 MCL tumor tissues investigated by genomic profiling [52, 53].

Therefore, unmutated MCL should result sensitive to ibrutinib therapy while mutations in the non-canonical NF-kB activation pathway could represent a resistance mechanism through BCR-pathway bypass.

Since it is estimated that 10–15% of MCL patients are *BIRC3*-mutated and that deletions (11q21-q23) involving *ATM* and *BIRC3* are quite common, it is plausible that *BIRC3* aberrations in MCL may result in decreased response to ibrutinib. This is due to the failure to suppress the alternative NF-kB pathway mediated by MAP3K14. This is in fact proposed as a therapeutic target in *BIRC3*‐mutated lymphomas and is currently under investigation [54].

### Glioma, glioblastoma and medulloblastoma

Gliomas are the most common tumor of the brain in humans. Overall, they are the most common primary malignant intracerebral neoplasm with an incidence rate of 6.03 per 100,000 individuals every year [55]. Low-grade gliomas (LGG) have an indolent course but may evolve to high-grade gliomas (HGG, like glioblastomas) that are aggressive malignancies [56].

Glioblastoma multiforme (GBM) is an aggressive form of tumor of the central nervous system. It is characterized by therapy resistance insurgence, associated to the disease recurrence. Surgery, radiotherapy and chemotherapy represent the main treatment options even though the prognosis remains dismal [3].

The evasion from apoptosis is a common strategy adopted by most tumors, including glioblastomas, and IAPs are among the most studied molecular and therapeutic targets also in these cancers. *BIRC3* (cellular IAP2) is one of the eight members of the human IAPs family [3, 12]. Tumor cancer genome atlas (TCGA) data analysis recently unveiled that *BIRC3* was specifically the only IAP whose differential expression was significantly related to the 5-year survival in patients with GBM. Lower *BIRC3* expression levels were associated to a favorable outcome and *BIRC3* levels-increase paralleled the acquisition of radio- and chemio-resistance.

IAPs members are also under investigation because responsible for the malignant progression of low-grade gliomas to glioblastomas. The TCGA analysis aimed at comparing the differential expression of LGG versus HGG unveiled that *BIRC3* is overexpressed in HGG and correlates with shorter PFS and OS in both the subtypes [56]. In the same research, matched samples also show that the expression increase of *BIRC3* characterizes the HGG who progressed starting from LGG. In vivo mouse glioma models also point to a role for *BIRC3* in promoting malignant progression of LGG towards HGG [56]. Malignant progression is the key event that transforms a LGG (with a PFS of years) in a HGG (having an expectancy of months). For this reasons, IAPs represent exceptionally potential therapeutic targets.

### Breast cancer

Breast cancer is one of the tumor types where *BIRC3* has not yet fully characterized. In this setting, *BIRC3* is regarded as an oncogene with antiapoptotic functions, at the same fashion of *Bcl2L1*, *Bcl2A1*, *RelB*, *Bcl3* and *MDM2* [57, 58]. *BIRC3* results also upregulated by the administration of the inflammatory cytokine 1β (IL-1β) to MCF-7 breast cancer cell line [59]. In this setting, *BIRC3* resulted the most up-regulated target in a panel of pro-survival genes. It was also linked to doxorubicin resistance after stimulation with the inflammatory IL-1β, strengthening its role as an oncogene in this type of cells.

A triple-negative cohort of breast cancer patients was investigated through the HTG EdgeSeq system combining a proprietary quantitative nuclease protection assay (qNPA) chemistry with Illumina next-generation sequencing (NGS) platform [60]. In this research, one of the genes upregulated in the sentinel or auxillary lymph nodes metastasis compared to the primary breast cancer was *BIRC3*, together with anti-apoptosis, survival signaling and chemotaxis genes.

The administration of anti-cancer agents, like the withanidolide Withaferin-A, has been demonstrated to cause the downregulation of XIAP, cIAP2 (*BIRC3)* and Survivin while inducing apoptosis of human breast cancer cells. The ectopic expression of these same anti-apoptotic mediators significantly inhibited the withaferin A-induced apoptosis [61]. Furthermore, inhibitors derived from the N-terminus tetrapeptide of Smac were able to antagonize cIAP1 and cIAP2 by binding their BIR3 domain. One of these (GDC-0152) in particular was efficient in reducing the tumor growth in breast cancer xenografts murine models [62].

However, not all the evidences are consistent with this postulated role, and one of the most recent papers reports an analysis based on the data of the Genomics of Drug Sensitivity in Cancer (GDSC) database. In this analysis emerges how *BIRC3* high expression correlate with sensitivity to kinase inhibitors, mostly targeting the ERK-MAPK pathway. The inhibitors included in the analysis are Selumentinib, Trametinib, Refametinib and (5Z)-7-Oxozeaenol. The group of cancers including breast invasive carcinoma was called “ERK-MAPKi sensitive group” [2].

### The BIRC3-paradox

The expression levels and tissue distributions of the IAPs members attracted the attention of many scientists involved in cancer research. The aim was (and still is) to develop and describe novel targeted anti-cancer compounds.

Genomics studies based on the Tumor Cancer Genome Atlas (TCGA) revealed how 7 IAPs members expression, including *BIRC3*, are distributed among 32 different types of cancers [2]. These proteins are also involved in a plethora of functions beyond apoptosis, like regulation of immune response, cell cycle, gene expression and DNA damage repair.

IAPs members are mostly involved in the regulation of the intrinsic (mitochondrial) and extrinsic apoptotic pathways, and to lesser extent in the execution phase of apoptosis. *BIRC3* and *BIRC6* are the ones involved in the regulation of at least one of these pathways in 78% and 69% of the types of cancers, respectively [2]. Interestingly, the small cohort of diffuse large B-cell lymphomas included in the analysis does not reveal any apoptotic gene regulated by *BIRC3*. Likewise, in LGG and breast invasive carcinomas *BIRC3* does not regulate any of the three pathways (intrinsic, extrinsic, execution phase). Moreover, neither *BIRC2* nor XIAP (*BIRC4*) are involved in apoptosis regulation of DLBCL and LGG, confirming the tight connection of these three IAPs. Interestingly, *BIRC2*, *BIRC3* and XIAP expression are also clustered together across the different tumors. When comparing tumor samples with their adjacent normal tissue (total of 32 human cancers), *BIRC3* results differentially expressed in a cancer-type specific manner. Likewise, *BIRC2* and XIAP have the same behavior [2].

At this point, scientists could face a paradox and, given the great potential of Smac-mimetics, should clarify the role of *BIRC3* in the tumor cell (Fig. 2). The presented results for the tumors of the central nervous system are different and bear different significance compared to the ones obtained on CLL. As we described, the transition from LGG to HGG is accompanied to an increase of *BIRC3* expression and a reduction of PFS and OS [56]. At variance with what described in gliomas, *BIRC3* genetic inactivation due to deletions and/or point mutations is a negative prognostic factor and one of the drivers of therapy-resistance insurgence in CLL patients [36, 38, 42, 43]. Other lymphomas, including MCL, rely on NF-kB activation and *BIRC3* disruption play a role in their pathogenesis. Smac-mimetics are also under investigation for therapeutic purposes [53, 54].

Shall *BIRC3* downregulation in the tumor cells regarded as a positive fact? Are *BIRC3*-interfering approaches advisable and represent a therapeutic opportunity for certain types of tumors, as the data on LGG and HGG demonstrate? Or, on the contrary, since *BIRC3* inactivation due to deletion, mutation or both is a negative, unfavorable marker (as CLL demonstrates and MCL suggests), the above-mentioned strategies should be avoided?

It becomes of primary importance to define whether *BIRC3* should be regarded as a proto-oncogene or, on the contrary, a tumor suppressor. This shall be done for each type of tumor, given the known existing differences. Pre-clinical experiments and clinical trials would greatly benefit of this elucidation.

## The role of BIRC5 during evasion of cancer cells from apoptosis

*BIRC5* is a very promising and well-studied therapeutic target because of its preferential expression in tumor cells of adult individuals. Here we review some of the most relevant evidences about the role of *BIRC5* emerged in hepatocellular carcinomas, gastrointestinal stromal tumors, prostate tumors and tumors of the nervous system.

Its expression in tumors belonging to various histological origins makes this IAP a pan-cancer druggable target (please, see Table 1).

### BIRC5 (survivin)

**Fig. 4:**
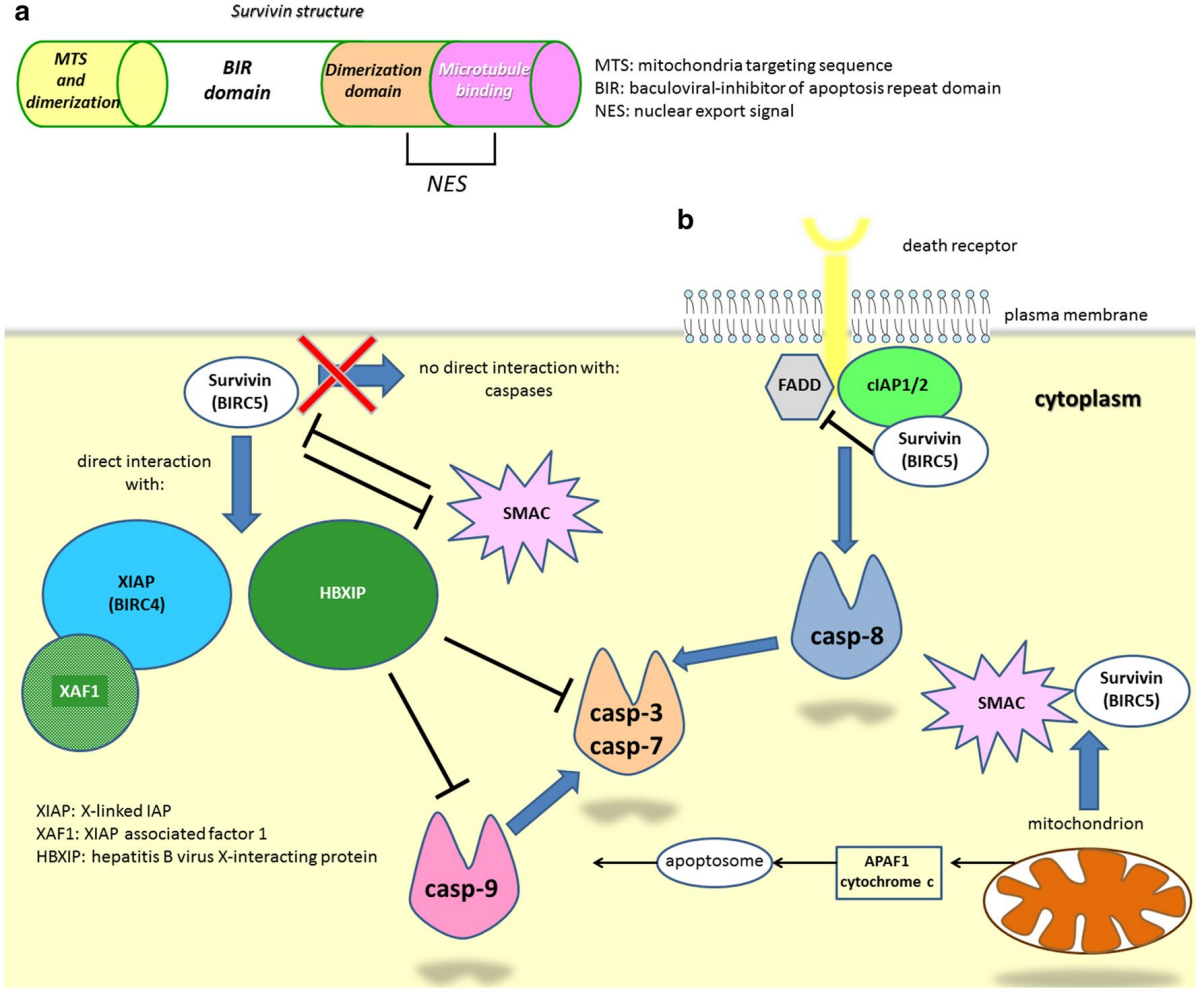
Survivin and interaction with apoptotic pathways. **a** Schematic representation of Survivin domains and their function. **b** Survivin interferes with the extrinsic apoptotic pathway through the inhibition of the death receptor/FADD-mediated signaling and the indirect inhibition of casp-8. Survivin also interacts with XIAP, XAF1 and HBXIP in a complex able to inhibit both the initiator casp-9 and the effectors casp-3 and casp-7

In order to assess whether the heterogeneity of information concerning *BIRC3* was common to other members of IAP family, we focused on another well-described mediator: *BIRC5* (survivin). *BIRC5* is one of the eight human IAPs genes and the best characterized of this family thus far (Fig. 4). Survivin is expressed by developing tissues, becomes undetectable in adult cells (except some specific cell types) and is re-expressed in tumors, where it has been reported to be highly present in lung, pancreatic, breast, ovarian, brain, colon cancer, among others [63–65]. In recent years, survivin pro-oncogenic role was described for many other tumors, including B-cell acute lymphoblastic leukemia, B-cell lymphoma and T-cell leukemia/lymphoma [66–68].

Hepatocellular carcinoma (HCC) is characterized by a low early detection rate, rapid progression and high recurrence rate. According to epidemiological data, HCC incidence is increasing in some Western countries like North America and Europe [69]. A gene-signature based on the analysis of differentially expressed genes (DEGs) from the Gene Expression Omnibus (GEO) and International Cancer Genome Consortium (ICGC) datasets was recently published [70]. In this work by Wang et al. [70], 276 differentially expressed genes characteristic of HCC compared to non.-cancerous tissues were identified. Univariate Cox regression analysis aimed at selecting prognostic genes yielded 10 genes defined as “hub” and identified by lowest p values. *BIRC5* emerged as one of the top scorer being among the four with the greatest prognostic value and its high expression significantly correlated to a lower patients survival.

High-risk gastrointestinal stromal tumors (GIST) rely on the genes belonging to the Wnt/β-catenin signaling pathway. The genes belonging to or activated by this pathway resulted upregulated in the high-risk group GIST and included *BIRC5*, among others [71].

Prostate cancer is another major health concern where *BIRC5* has been studied and described. Since p53 transcriptionally represses *BIRC5* expression in normal tissues, mutations affecting p53 cause *BIRC5* upregulation [72]. A significant subset of prostate cancer patients display mutated p53 and is characterized by tumor aggressiveness and significantly increased risk of progression after radical prostatectomy [73]. Recent data obtained on a very large number of prostate cancer samples demonstrate that *BIRC5* mRNA increased in prostate cancer and prostate cancer metastases compared to tissues from healthy donors or from adjacent normal prostate tissues combined [74]. Interestingly, from the same study emerges that cytoplasmic localization is associated to an aggressive disease (higher Gleason score), higher pathological tumor stage and higher Ki67 proliferative index. This is consistent with the fact that cytoplasmic *BIRC5* originates from mitochondria and it is rapidly released from them upon pro-apoptotic stimuli. *BIRC5* in the cytoplasm then interacts with XIAP (also upregulated in prostate cancer). The resulting heterodimers facilitate anti-proteasomal stability and inhibition of caspase-mediated apoptosis, thereby promoting tumor growth and survival (Fig. 4) [74].

Survivin plays mitosis regulatory function and anti-apoptotic function in gliomas, and it has been reported to be localized both in the nucleus and in the cytoplasm consistently [64]. However, many immunohistochemical (IHC) results were obtained with different antibodies or were analyzed differently (different choices of thresholds), thus lack of standardization still represents an issue.

Furthermore, 12 total studies compared glioma grade with survivin expression and 8/12 reported an association while 4/12 did not report any. Likewise, the attempt to correlate glioma survival with survivin expression by IHC led to inconsistent results, possibly due to the heterogeneity of cut-offs and analysis methods applied to categorize survivin levels in glioma patients [64].

A recent study conducted on 133 formalin fixed-paraffin embedded (FFPE) diffuse astrocytic tumors of three different subtypes reports a correlation between survivin, tumor subtypes and patients survival [75]. IHC staining reveals that high p53 expression and survivin nuclear localization correlate with the anaplastic astrocytoma whereas the cytoplasmic localization of survivin correlate with the glioblastoma subtype. Regardless of the subcellular localization, the high survivin and p53 expression correlate with a lower short-term and long-term survival of the patients, who would benefit of radiotherapy [75]. These data appear consistent with the postulated role of survivin, but definitely unexpected when referring to the well-established tumor suppressor role of p53. This apparent paradox can be explained by the fact that IHC data represent just the semi-quantitative expression and localization of the protein, but do not take into account the activation status of p53. Actually, it is well established that p53 activity is modulated also through post-translational, epigenetic modifications. Therefore, a high p53 protein level does not necessarily correspond to a highly activated oncosuppressor.

Survivin has been reported to promote cancer cell proliferation in a variety of tumors, including gliomas. A model system of glioma cells overexpressing survivin has been developed by Conde et al. [76]. Interestingly, survivin overexpression led to the significant increase of numerical and structural chromosomal aberrations compared to the karyotype of the mock cell line. The chromosomes gains or losses were also significantly different in the survivin-overexpressing cells. This demonstrates a role for this IAP in increasing chromosomal instability [76].

Another recent research involved 131 patients with a histopathological diagnosis of astrocytic tumors (diffuse astrocytoma, anaplastic astrocytoma and glioblastoma). IHC was used to detect caspase-3, survivin and MIB-1 expression [77]. Although no molecular investigation was carried out in this manuscript, the presented results are consistent and show that caspase-3 was expressed in all the 31 primary glioblastomas but only in 17/30 (56.7%) secondary glioblastomas. Survivin expression, on the contrary, was observed in 80.6% primary glioblastomas and in all the examined secondary glioblastomas. These data support the anti-apoptotic role of survivin.

Medulloblastomas (MB) are aggressive tumors of the brain occurring primarily in children. It was demonstrated already several years ago that high survivin expression associates to MB malignancy and poor-prognosis and this IAP-member is recognized as a therapeutic target with high priority in these subtypes [78].

Later on, a very informative mouse model of Sonic Hedgehog signaling-driven MB (SHH-MB) was created and served to elucidate the molecular role of survivin in MB cells [79]. SHH-MB tumors and granule neuron precursors displayed high-survivin expression, at variance with normal adult cerebellum. Importantly, cell proliferation upon genetic disruption is dramatically reduced, as demonstrated by a 90% reduction in radioactive-thymidine incorporation [79]. Cell cycle progression is also impaired upon disruption, demonstrating a survivin essential role in the processes leading to cell division. In order to translate these results, patient-derived xenografts (PDXs) from SHH-driven tumors were treated with specific antagonists that were able to inhibit SHH-driven MB cell growth. Finally, the authors show the *in vivo* MB growth inhibition by survivin antagonists in mouse models, demonstrating the phenotypic effects of the inhibition of this single IAP in MB. The double effect of perturbing cell cycle and promoting cell death by apoptosis makes survivin a promising therapeutic targets also in this setting.

All these evidences explain, at least in part, the already known chemo-and radio-sensitizing effects of survivin silencing and inhibition, demonstrated in a variety of cancers either solid or haematological [80–83].

The mechanisms for survivin inhibition encompass various layers of regulation. All the documented inhibitors can be classified into 5 categories, based on the characterizing mechanism of action: (a) Inhibitors that disrupt survivin interactions with its partner proteins; (b) Inhibitors that disrupt survivin homodimerization; (c) Inhibitors that decrease survivin gene transcription; (d) Inhibitors that induce survivin mRNA degradation; and (e) survivin or its peptide for immunotherapy [84].

Several upstream regulators are known and involve a plethora of pathways, recently summarized by Li et al. [85] based on the Gene Ontology (GO) database (https://portal.genego.com/). Some of the survivin inhibitors (e.g. SMAC-mimetic UC-112) feature a selectivity that promotes proteasome-mediated degradation of this IAP, leaving the other members (XIAP, cIAP1, cIAP2 and Livin) mostly unaffected. Others are, on the contrary, effective towards more than one member of the family. All of the several documented molecules share the characteristics of being aimed at impairing and weakening survivin functions in the various cancer experimental models. Thus, at variance with *BIRC3*, survivin is considered as a pro-oncogenic protein and is commonly targeted to impair tumor cell proliferation. It has to be mentioned though that splice variants (DEx3 and 2B) have been reported to have specific roles in some tumor cells and that the existence of splice variants represents another layer of regulation for *BIRC5* [86, 87].

## Concluding remarks

The concept arising from the recent literature is that the biological meaning of *BIRC3* disregulation in cancer cells is not entirely predictable. The disregulation may be genetic (deletions, insertions, point mutations), transcriptional, or a combination of these two levels. Some conflicting experimental data reporting either the oncogenic or the tumor suppressor role in the same type of malignancy strengthen the context-dependent and bi-faceted role of *BIRC3*.

The available data concerning *BIRC5* are, on the contrary, rather homogeneous and point to a tumor promoting role for this IAP.

Cancer cells of different histological origin rely on different pathways to survive and proliferate and *BIRC3*, in each type of cancer, may interact and contribute at different levels. This leads to the conclusion that the application of a given IAP-inhibitory therapeutic strategy (e.g. SMAC mimetics, interfering approaches, etc.), should be preceded by the evaluation of the survival and proliferation pathways that malignancy relies on.

## Data Availability

All data generated or analysed during this study are included in this published article [and its supplementary information files].

## Abbreviations

IAPs: Inhibitors of apoptosis proteins
BIR: Baculoviral IAP repeat domains
UBC: C-terminal ubiquitin-conjugating domain
CARD: Caspase recruitment domain
RING: C-terminal Ring zinc-finger domain
BIRC: Baculoviral IAP repeat containing
XIAP: X-linked IAP
SMAC/Diablo: Second Mitochondria-derived Activator of Caspases/Direct IAp Binding with Low pI
DLBCL: Diffuse large B-cell lymphoma
CLL: Chronic lymphocytic leukemia
MCL: Mantle cell lymphoma
AML: Acute myeloid leukemia
cIAP: Cellular IAP
OSCC: Oral squamous cell carcinoma
PFS: Progression free survival
OS: Overall survival
FCR: Fludarabine-cyclophosphamide-rituximab
BTK: Bruton tyrosine-kinase
del11q-: Deletion of 11q
BCR: B-cell receptor pathway
LGG: Low-grade gliomas
HGG: High-grade gliomas
GBM: Glioblastoma multiforme
IL-1β: Inflammatory cytokine 1β
TCGA: Tumor cancer genome atlas
GDSC: Genomics of Drug Sensitivity in Cancer database
IHC: Immunohistochemical
FFPE: Formalin fixed-paraffin embedded
MB: Medulloblastomas
SHH-MB: Sonic Hedgehog signaling-driven MB
PDXs: Patient-derived xenografts
NPC: Nuclear pore complexes
SINE: Selective inhibitors of nuclear export
DEGs: Differentially expressed genes
GEO: Gene Expression Omnibus
ICGC: International Cancer Genome Consortium
